# Platinum-Nucleos(t)ide Compounds as Possible Antimetabolites for Antitumor/Antiviral Therapy: Properties and Perspectives

**DOI:** 10.3390/pharmaceutics15030941

**Published:** 2023-03-14

**Authors:** Federica De Castro, Erika Stefàno, Erik De Luca, Michele Benedetti, Francesco Paolo Fanizzi

**Affiliations:** Dipartimento di Scienze e Tecnologie Biologiche ed Ambientali, Università del Salento, Prov.le Lecce-Monteroni, Centro Ecotekne, 73100 Lecce, Italy

**Keywords:** nucleoside analogues, platinum compounds, coordination compounds, antitumor drugs, antiviral drugs, antimetabolites

## Abstract

Nucleoside analogues (NAs) are a family of compounds which include a variety of purine and pyrimidine derivatives, widely used as anticancer and antiviral agents. For their ability to compete with physiological nucleosides, NAs act as antimetabolites exerting their activity by interfering with the synthesis of nucleic acids. Much progress in the comprehension of their molecular mechanisms has been made, including providing new strategies for potentiating anticancer/antiviral activity. Among these strategies, new platinum-NAs showing a good potential to improve the therapeutic indices of NAs have been synthesized and studied. This short review aims to describe the properties and future perspectives of platinum-NAs, proposing these complexes as a new class of antimetabolites.

## 1. Introduction

Nucleos(t)ide analogues (NAs) antimetabolites are drugs structurally similar to either purine and pyrimidine bases or to the corresponding nucleos(t)ides [1,2,3,4,5]. In this context, the folate cofactors are also considered antimetabolites, because they are involved in several steps of the purine and pyrimidine biosynthesis [6,7]. Drugs of this class have been developed for more than 50 years and, nowadays, are commonly used in the treatment of cancers and viral diseases [2,4,6], Table 1. These antimetabolites act by interfering with the production or function of nucleic acids, RNA and DNA, and preventing cells’ growth and/or survival, since biological systems cannot generally use them in a productive manner [8].

In recent years, for antitumor/antiviral activity, pharmaceutical industries have prepared and evaluated several novel drugs based on nucleos(t)ides analogues (NAs), confirming the ongoing and also applicative interest for this important research line. The increasing knowledge of the physiological mechanisms operating in viral and cancer diseases development and progress helped the design of new active NAs to be addressed against specific biological targets.

**Figure 1:**
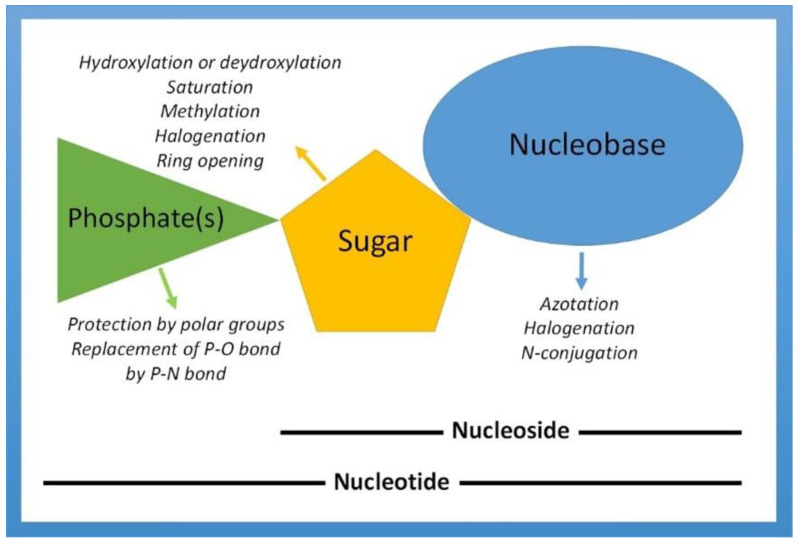
Schematic representation indicating the most common chemical modifications of nucleobases, nucleosides, and nucleotides suitable to give pharmacologically active antitumor and/or antiviral analogues. Adapted from [2], published by Nature Publishing Group, 2013.

The structural and chemical modifications of NAs are generally produced by *N*-conjugation or azotation and halogenation for the nucleobase, and by hydroxylation or dihydroxylation, methylation, halogenation, ring opening, and saturation for the ribose moiety [2]. In the case of nucleotides, the protection by polar groups or replacement of P-O bonds by P-N bonds may also occur, as shown in Figure 1.

Despite chemical changes, showing a structure very similar to that of endogenous nucleos(t)ides, the NAs can maintain the ability to enter cells by exploiting specific plasma membrane nucleoside transporters [34,35]. Organic cation and/or anion transporters, not specific for nucleosides, can also be involved in the cellular uptake of some NAs [2]. These classes of antitumor and/or antiviral compounds can differ for the types of transporters used and for their preferential interaction with specific targets. This can explain the effectiveness differences observed for these compounds in the treatment of neoplasia with protracted evolution, rapidly proliferating tumors, and viral diseases [36]. A major difference of the NAs used as antiviral drugs, with respect to those used as antitumor agents, consists in a generally lower affinity for the mammalian enzymatic systems. Another, but not secondary, aspect is the lower dosages generally required to produce an effective antiviral activity compared to those necessary for a relevant antitumor activity. Both these characteristics concur in determining a generally better tolerance profile when the NAs are used as antiviral drugs rather than as antitumors.

**Figure 2:**
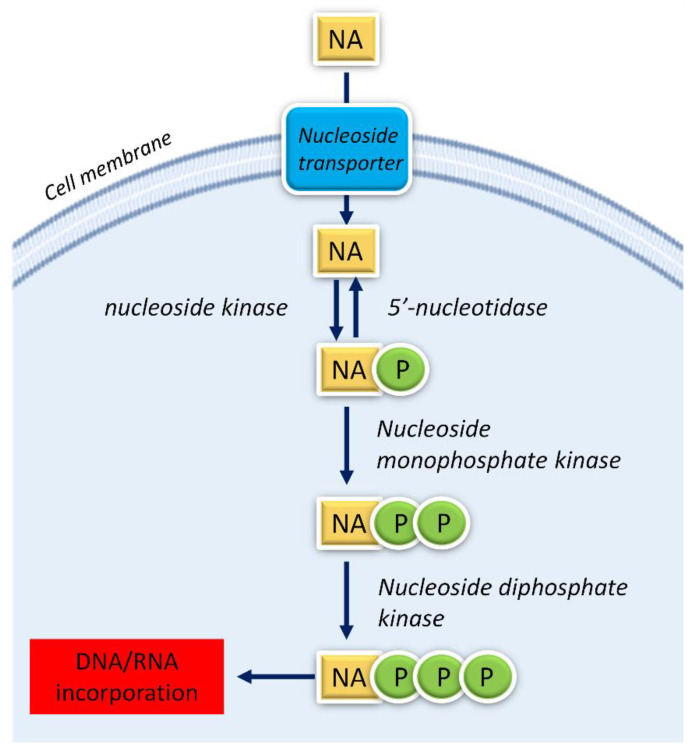
Mechanism of action of NAs. The NAs generally enter cells crossing the cell membrane by an active transport mechanism operated by plasma membrane transporters. Once inside cells, the NAs are progressively phosphorylated and so produce the final triphosphate derivatives.

All NAs use similar mechanisms of action to exert their activity. They enter the cells through an active transport across the cell membrane by using specific membrane transporters. After entering the cells, NAs use the same metabolic pathways of the endogenous nucleos(t)ides [37]. Their mechanism of action includes a first step of phosphorylation, catalyzed from the nucleoside kinase, undergoing production of the nucleoside monophosphate analogue. Two further phosphorylation steps, performed, respectively, by nucleoside monophosphate kinases and nucleoside diphosphate kinases, produce the active triphosphate form [38] (see Figure 2). Cellular or viral nucleic acids can incorporate the triphosphate forms of nucleoside analogues which compete with their counterparts, eventually inhibiting essential enzymes such as human and viral polymerases, therefore interfering with cancer cell growth or viral replication [2].

As for most of the anticancer drugs used in chemotherapy, and also for NAs, some mechanisms of resistance may occur. The main causes of resistance to NAs in both cell lines and clinical samples are (i) inefficient cellular uptake and subsequent deficient intracellular concentration of the NAs triphosphates; (ii) prevented DNA strands and/or dNTP pools damaging; and (iii) faulty apoptosis induction [36,39].

The development of novel drugs candidates able to reduce the resistance, improve the oral bioavailability and overcome the inter-individual variability (which requires a dose adaptation) is still highly desired. One of the employed therapeutic approaches consists in combining the NAs therapy with other agents modulating the apoptotic responses. An example is combination chemotherapy. This approach, adopted for high rate malignant tumors, takes advantage of the combined use of different therapeutic agents, providing additional benefits to the treatments [36]. A multifactorial approach may allow significant progress in the treatment of cancer and viral diseases. In 1997, Mosconi et al. reported the effectiveness of combining gemcitabine and cisplatin in non-small cell lung cancer. By combining gemcitabine, a pyrimidine analogue, with cisplatin, one of the most potent anticancer agents used in the treatment of various solid tumors, the response rates significantly increased with respect to either drug used alone [40]. Nowadays, this combined treatment (cisplatin–gemcitabine) is highly recommend for the treatment of advanced (metastasis) non-small cell lung cancer [41].

In this context, metal-based drugs have shown great flexibility in their potential ranging from anticancer [42,43,44] to antiviral applications [45,46] and several other disorders [47,48]. Differences in ligands coordination to metal may have a great impact on their efficacy, strongly affecting pharmacodynamics and pharmacokinetics profiles. Metallodrugs are often prodrugs which undergo activation by ligand substitution or redox reactions. Both essential and non-essential metals are used in drug therapy and diagnosis [47,48]. Some of the first metallodrugs used in therapy were antimicrobial and antiparasitic agents based on arsenic. In fact, in 1912, Paul Ehrlich and co-workers discovered salvarsan, an arsenic-based drug effective against syphilis, signing the birth of modern chemotherapy [49]. Nowadays, melarsoprol, despite its severe side effect of encephalopathy, is an approved arsenic drug that is still used against trypanosomiasis. Two other types of antimicrobic and antiparasitic drugs are represented by antimony(III) and (IV) metallodrugs (e.g., sodium stibogluconate and melglumine antimoniate), which act against cutaneous and mucocutaneous leishmaniasis [48]. While arsenic- and antimony-based drugs showed high toxicity compared to their therapeutic benefit, bismuth is non-toxic and well tolerated, even at high doses [48]. Bismuth drugs are utilized as a broad-spectrum antibiotic; they have been demonstrated to be effective against antibiotic-resistant bacteria (e.g., Helicobacter pylori) thanks to their ability to bind and inhibit key enzymes such as metallo-β-lactamase (MBL) [50]. Bismuth subcitrate (CBS) or ranitidine bismuth citrate (RBC) are used for the treatment of peptic ulcers that are often associated with H. pylori, while tribromophenate–bismuth(III) (Xeroform) is an antimicrobial agent applied in the treatment of chronic wounds. Again, bismuth subsalicylate (BSS) is a widely used metal-based drug for the treatment of gastrointestinal disorders.

Complexes of copper, gold, and silver have been used extensively, since they demonstrate anticancer, anti-inflammatory, antimicrobial, and antiparasitic activity [51,52]. Silver sulphadiazine is an example of a silver-based drug approved for the prevention and treatment of infections resulting from second- or third-degree burns. On the other side, gold compounds, mainly gold(I) cyanide and thiosulfates, were introduced and approved for rheumatoid arthritis treatment. Among these, we might mention sodium aurothiomalate, aurothioglucose, sodium aurothiopropanol sulfonate, sodium aurothiosulfate, and the much newer gold(I) compound, auranofin (tetraacetyl-β-D-thioglucose-gold(I)-thioethylphosphine), approved by the FDA in 1985. These types of metallodrugs are defined as disease-modifying antirheumatic drugs (DMARDs), being able to slow the progression of rheumatoid arthritis by inhibiting several cathepsins implicated in these diseases [48].

Some metal-based drugs were applied for other types of disorders. For example, the superoxide dismutase-mimicking macrocycle (M40403) is a promising manganese(II) metallodrug candidate for the treatment of cardiovascular disorders [48]. Again, lithium-based drugs represent modern psychopharmacological agents and are the gold standard in the treatment of bipolar disorders and mania [53,54]; a lithium carbonate formulation was first approved in 1979, while a lithium citrate tetrahydrate is currently undergoing clinical trials for the treatment of Huntington’s disease [48].

An important step in the development of metallodrugs was the discovery of cisplatin and its clinical debut in 1978 [55]. Platinum drugs are used in 50% of cancer chemotherapies. Together with cisplatin, carboplatin and oxaliplatin are the only FDA-approved platinum drugs, but there are other platinum complexes which have been approved in single nations, such as nedaplatin, heptaplatin, lobaplatin, miriplatin, and dicycloplatin [56,57]. The mechanism of platinum-based anticancer drugs involves the formation of platinum-DNA adduct and inter- and intra-DNA strand cross-links, which interfere with DNA replication and DNA transcription, causing cell cycle arrest and cell apoptosis/necrosis [58]. Furthermore, platinum complexes have also demonstrated to be good antiviral and antibiotic agents and protect cells from different types of viruses and bacteria [59,60].

Focusing on their applications as anticancer or antiviral, antivirals metal-based drugs can block viral infections, relieve symptoms, regulate the human immune system, and inhibit virus replication enzymes [47]. When used as adjuvants, they can also improve the effectiveness of antiviral drugs and vaccines [61]. Anticancer metal-based drugs can act via metal–DNA or metal–protein adducts formation (thus, through a genomic or non-genomic target), then activating specific mechanisms leading to cancer cell death [62,63,64].

This review aims to highlight the properties and the potential of platinated nucleos(t)ides-based compounds, which may represent a new promising class of antimetabolites with merged properties between those of platinum compounds and those of nucleoside analogues. The perspectives in the treatment of cancer and viral diseases are discussed here.

## 2. Platinated Nucleos(t)ides

**Figure 3:**
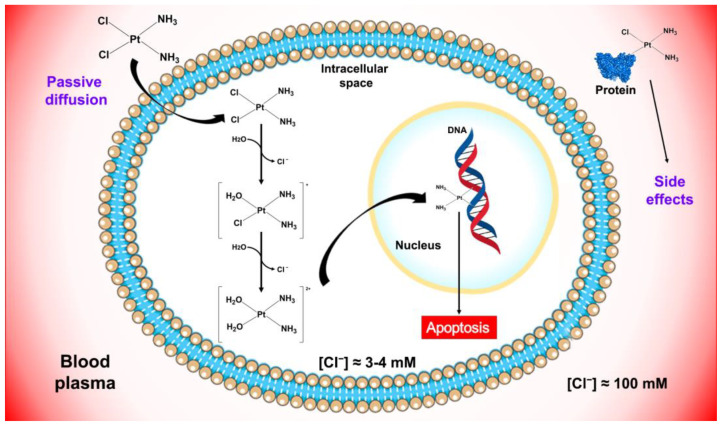
Schematic representation of cisplatin mechanism of action.

Metal-based antitumor drugs constitute a very successful class of drugs. In this class, the main clinical and therapeutic successes can be referred to the wide family of platinum-based antitumor drugs, starting from the first discovered platinum-based drug named cisplatin [65]. After cisplatin discovery, several platinum complexes have been synthesized but only few of them have been successfully marketed worldwide (e.g., cisplatin, carboplatin, oxaliplatin) as chemotherapeutic drugs [44,66,67,68,69,70]. Cisplatin induces cytotoxic effects through binding DNA, thus interfering with the physiological DNA transcription and/or replication [71,72,73] (see Figure 3).

Platinum compounds act as antiviral agents by interfering with DNA or RNA synthesis to exploit their mechanism of action. Moreover, it has recently been demonstrated that they are able to induce the virus’s death without affecting the host cell [74].

Starting from the wide research interest on platinum drugs as chemotherapeutic agents, thousands of new platinum compounds were synthesized [65,75,76,77,78]. Considering the high reactivity of platinum complexes with the DNA nucleobases, some new platinum-nucleoside analogues were synthesized [79]. These can be considered as special nucleobase analogues, where the modification of the nucleoside mainly consists of a coordination to a metal complex.

Here, we highlight the demonstrated antitumor and antiviral activities of platinated nucleos(t)ides, confirming the adaptability of this class of complexes to the different fields of application (as anticancer and/or antiviral drugs).

### 2.1. Anticancer Activity

Despite the great progress made in oncology research, the lack of selectivity towards tumors and the high systemic toxicity of many approved drugs make this research field of great interest in the scientific community. The main objectives are focalized on the search for new drugs with a better bioavailability and fewer side effects.

**Figure 4:**
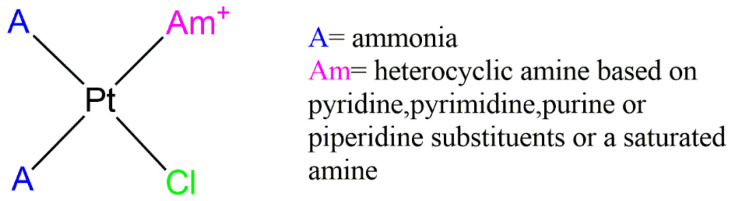
Chemical structure of cationic triamine complexes. A = ammonia; Am = heterocyclic amine based on pyridine, pyrimidine, purine, piperidine, or saturated amine.

In 1989, Hollis et al. prepared and screened 34 cationic compounds against in vivo murine tumor models [80]. They proposed a series of platinum antitumor agents violating the classical structure–activity relationships, being cationic Pt(II) complexes with three coordinated *N*-donor ligands, as reported in Figure 4. Among these compounds, some platinum(II)-nucleoside complexes of the type *cis*-[Pt(NH_3_)_2_(Am)Cl]^+^ demonstrated high stability and solubility in aqueous media and an interesting antitumor potential against different tumors, including Sl80a, P388, and L1210 in mice [80].

In detail, *cis*-[Pt(NH_3_)_2_(*N3*-cytosine)Cl]Cl and *cis*-[Pt(NH_3_)_2_(*N7*-2′-deoxyguanosine)Cl]Cl complexes showed the best combination of activity and potency in the S180a screen. In the L1210 leukemia screen, *cis*-[Pt(NH_3_)_2_(*N3*-cytosine)Cl]Cl and *cis*-[Pt(NH_3_)_2_(*N3*-cytosine)Cl]Cl demonstrated good antitumor activity. *Cis*-[Pt(NH_3_)_2_(*N3*-cytosine)Cl]Cl was also found active against P388 leukemia.

**Figure 5:**
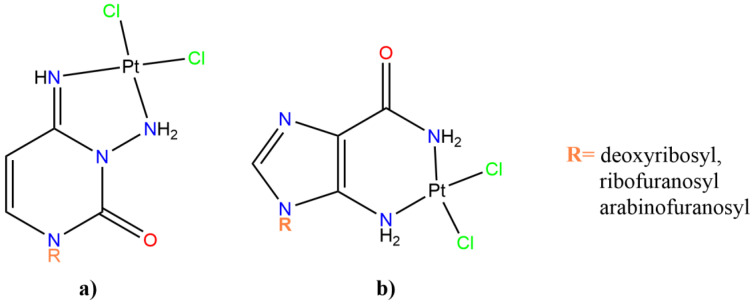
Schematic representation of the *cis*-dichloridoplatinum(II)-*N*-aminated nucleoside complexes. (**a**) Pt-cytosine analogues; (**b**) Pt-guanosine-like derivatives. R = deoxyribosyl, ribofuranosyl, arabinofuranosyl.

Other *cis*-dichloridoplatinum(II)-*N*-aminated nucleoside complexes (see Figure 5) were synthesized and tested for their antitumor effect against L1210 in vivo (in mice) and in vitro by Maeda et al. [81].

**Figure 6:**
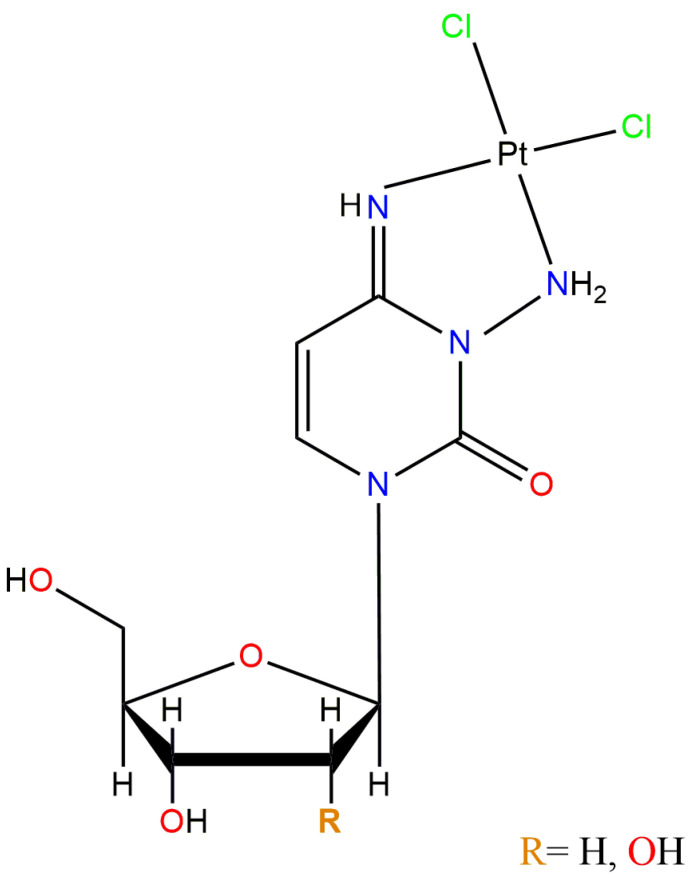
Chemical structure of 3-amino-2′-deoxycytidine-dichloridoplatinum(II) and 3-aminocytidine-dichloridoplatinum(II).

For all these types of complexes, higher activity and a much lower nephrotoxicity in mice with respect to cisplatin was observed. Examples are the 3-aminocytidine-dichloridoplatinum(II) and 3-amino-2′-deoxycytidine-dichloridoplatinum(II) (see Figure 6), which possess high antitumor activity against L1210 cells in vivo in a wide dose range from 10 to 200 mg/kg. The in vitro cytotoxic effects in L1210 cells demonstrated higher cytocidal effects than the parent not-platinated *N*-aminated nucleosides. The IC_50_ values of the complexes were higher than that of *cis*-DDP by factors of 10 to 10^3^.

Extending the study on this class of *cis*-dichloridoplatinum(II)-*N*-aminated nucleoside complexes, researchers also established that their mechanism of action caused the inhibition of DNA and RNA synthesis, as occurs for widely used anticancer/antiviral antimetabolites. Maeda et al. demonstrated the role of sugar moiety in the action mechanism of the considered complexes: the Pt-nucleoside complexes having ribose inhibited RNA synthesis and those having deoxyribose or arabinose inhibited DNA synthesis [81].

**Figure 7:**
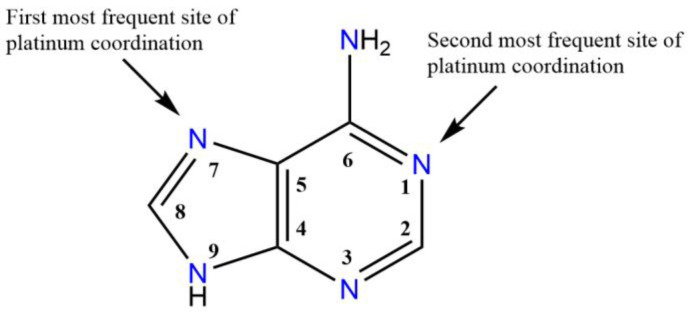
Structural formula of adenine, with the preferred coordination sites of platinum to nitrogen evidenced. The atom numbering scheme, for nucleotides, is also reported.

Recently, Štarha et al. offered an overview of platinum(II) and platinum(IV) complexes containing adenine as ligands or its derivatives coordinated to platinum through *N*- or *C*-donor atom(s) of the adenine moiety [82]. Many complexes of this type have been synthesized, demonstrating the interest of researchers in this new class of nucleoside analogues [83,84]. Complexes containing monodentate *N*7 coordinating adenine-based ligand(s) are the most numerous with respect to the *N6*, *N9*, *C8,* or bidentate *N1,N6,* which are relatively rare (see Figure 7).

Summaries of the known biological activities of tested complexes were also reported. For the [PtCl_2_(Ado-*N6*,*N7*)] complex, in vivo tests against mice Sarcoma 180 cells were performed by Cleare and Hoeschele in 1973. Unfortunately, for the mentioned compound, low activity was showed [85].

Baranowska-Kortylewicz et al. studied the in vitro cytotoxicity of new complexes of the type dichlorido(6-aminoethylaminopurine)platinum(II) and dichlorido(6-hydroxyethylaminopurine)platinum(II) against different carcinoma cancer cells, while also investigating nephrotoxicity in the mouse kidney [86]. Despite the lower cytotoxicity on tumor cells, an interesting, relatively lower renal toxicity was also observed compared to cisplatin.

**Figure 8:**
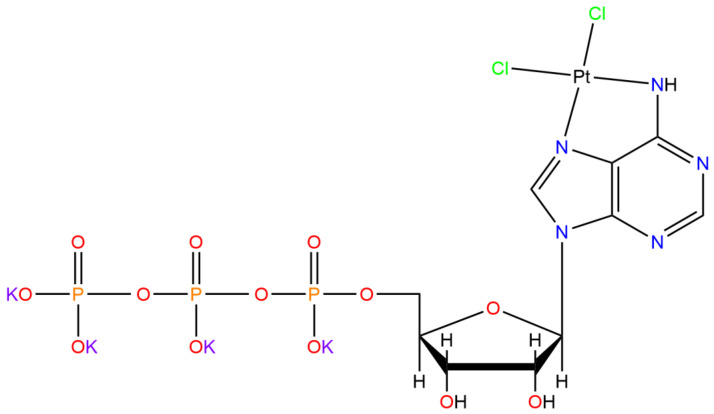
Chemical structure of the *cis*-K_4_[PtCl_2_ATP] complex, adapted from [10], published by Nature Publishing Group, 2020.

Other synthesized platinum complexes containing adenosine showed anticancer activity in in vitro assays. An example is the antineoplastic agent *cis*-K_4_[PtCl_2_ATP] synthesized by Nayak et al. (see Figure 8), which demonstrated active against Dalton’s lymphoma cells [87].

Kirschner et al. reported that platinum(IV) complex of the antimetabolite 6-mercaptopurine (mp), [Pt(mp_2_)Cl_4_], was effective against the murine tumors S180 (Sarcoma 180) [88,89]. In this study, researchers demonstrated that the platinum complex with 6-mercaptopurine as ligand showed a more enhanced anticancer activity than the free ligands. The used ligand, 6-mercaptopurine (see Table 1), is an antimetabolite with known antineoplastic and immunosuppressant properties [90]. It is generally used in combination with other drugs, inhibiting purine metabolism and, as consequence, the nucleic acid synthesis.

**Figure 9:**
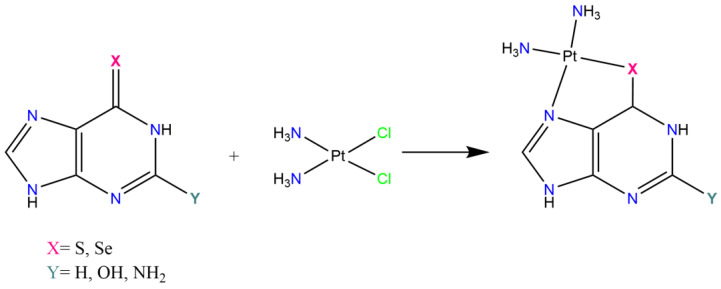
Schematic representation of the platinum complexes synthesized by Maeda et al.

Other thio and selenoguanine–platinum complexes were reported from Maeda et al. [89,91], as shown in Figure 9. In detail, aquated *cis*-diamminoplatinum(II) reacted in slightly acidic conditions with stoichiometric amounts of selenopurine or thiopurine and the resulting complexes were studied for their antitumor activity against L1210 cells in mice. These compounds exhibited a satisfying antitumor activity and very low toxicity. Although their mechanisms of action were not fully understood, the antitumor activity of these complexes seem to also be due to a slow release of the ligands, therefore resulting in a merged action of both the residual platinum complex moiety and the purine analogues.

One of the reasons for the continued study of platinum complexes is the search for new drugs able to circumvent the resistance phenomena that often occurs during cisplatin treatment. In addition to Pt(II) complexes, Pt(IV) complexes also showed high potential as anticancer drugs, even for their ability to work as prodrugs.

**Figure 10:**
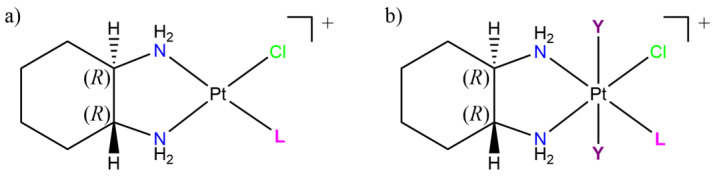
Chemical structures of model platinum(II) and (IV) nucleoside monoadducts of the type (**a**) [Pt(DACH)LCl]NO_3_ and (**b**) [Pt(DACH){*trans*-(Y)_2_}LCl]NO_3_, respectively. DACH = *trans*-*1R*,*2R*-diaminocyclohexane; L = adenine, guanine, hypoxanthine, cytosine, adenosine, guanosine, inosine, cytidine, 9-ethylguanine, or 1-methylcytosine; Y = OH, acetate.

Model platinum-nucleobase and -nucleoside complexes were synthesized and studied for their antitumor activity by Ali et al. The mentioned study concerns a series of platinum(II) and (IV) monoadducts of the type [Pt(DACH)LCl]NO_3_ and [Pt(DACH){*trans*-(Y)_2_}LCl]NO_3_, respectively. A total of 30 platinum monoadducts nucleobases complexes, where DACH = *trans*-*1R*,*2R*-diaminocyclohexane, L = adenine, guanine, hypoxanthine, cytosine, adenosine, guanosine, inosine, cytidine, 9-ethylguanine or 1-methylcytosine, and Y = hydroxo or acetate ligand (see Figure 10), were synthesized.

These platinum-nucleoside monoadducts may also have the ability to further interact with DNA. The cytotoxicity for some of the synthesized compounds against A2780 and A2780CP human ovarian cell lines that were, respectively, sensitive and resistant to cisplatin was also evaluated in comparison with the latter.

Although able to show cytotoxicity on cisplatin-resistant A2780CP cells, also in a low concentration range, the nucleobase adducts proved less effective against A2780 cells then cisplatin (4 to 29 fold). Consistently, cisplatin needs a higher concentration against A2780CP-resistant cells with respect to the sensitive, showing the used A2780CP strain, high levels of resistance (resistance factor of 44.4). Among the nucleobase derivatives, [Pt(DACH){*trans*-(acetate)_2_}(9-EtGua)Cl]NO_3_ proved the most potent and biologically active, with an IC_50_ of 1.1 and 3.23 µM on the A2780 and A2780CP, respectively. The authors also highlighted the ability of the complex to circumvent the cisplatin resistance [92].

### 2.2. Antiviral Activity

The appearance of new epidemic viruses requires the search for new effective antiviral drugs. Nucleoside and nucleotide analogues belong to the largest class of agents active as antivirals. These usually follow similar metabolic pathways as the endogenous nucleos(t)ides, creating interferences and alterations of the normal metabolic pathways [1,5,38,93,94,95]. In fact, they are widely used for the treatment of both acute and chronic viral infections since they may inhibit virus replication, by interfering with the synthesis of DNA and/or RNA in infected cells. Nucleos(t)ide analogues drugs generally cause the inhibition of specific intracellular enzymes (e.g., viral polymerases) acting as a substrate for the viral enzymes. In this way, nucleoside/nucleotide analogues can be incorporated into newly synthesized nucleic acids in competition with their endogenous congeners.

The incorporation of nucleoside or nucleotide analogues into DNA and/or RNA can lead to the termination of chain elongation or the occurrence of genetic mutations in the viral progeny [96]. The nucleoside analogues-based drugs can be based on structurally modified nucleos(t)ides, in the nucleobase and/or sugar moieties, with the aim to enhance the biological interactions able to generate antiviral activity [43]. In this optic, Acyclovir (ACV) was the first modified nucleoside-based drug effective for the therapeutic treatment of herpes virus (HSV) infections [97]. For its structural analogies with guanosine, ACV’s coordination ability toward metals generated a wide interest among researchers. Coluccia et al. first reported the synthesis of “hybrid drugs” based on the combination of *cis*-amine platinum antitumor compounds with antiviral nucleotide analogues [59]. In particular, they investigated the in vitro antiviral properties of *cis*-[Pt(*N7*-ACV)Cl(NH_3_)_2_]NO_3_ (*cis*Pt-ACV) on VERO cells infected with HSV-1. The *cis*Pt-ACV complex showed good antiviral activity against HSV-1, albeit less effective than acyclovir. This could be explained by the possible low affinity of the viral thymidine kinase and/or of the host cell kinases for the *cis*Pt-ACV complex compared to the simple ACV. More in-depth studies also showed that the incorporation of monofunctional *cis*Pt-ACV inhibits DNA synthesis and blocks RNA polymerases at platinated guanine residues [98]. Subsequently, Margiotta et al. studied four novel hybrid Pt-complexes containing the phenanthroline (phen) or neocuproine (Me_2_phen) aromatic diimines as platinum carrier ligands, and the antiviral nucleotide analogues ACV or penciclovir (PEN), coordinated to the metal through *N(7)*. In particular, the [Pt(ACV)_2_(phen)](NO_3_)_2_], [Pt(PEN)_2_(phen)](NO_3_)_2_, [Pt(ACV)_2_(Me_2_phen)]I_2_, and [Pt(PEN)_2_(Me_2_phen)](NO_3_)_2_ complexes were synthesized and tested against different strains of virus (HSV, HIV, CMV) [99,100]. It emerged from the analysis that ACV-containing compounds had antiviral activity similar to ACV against HSV-1 (strain KOS) and HSV-1 (TK-KOS-ACVr strain). Furthermore, they also seemed to be more effective than ACV against HSV-2. Indeed, the [Pt(ACV)_2_(Me_2_phen)]I_2_ compound showed antiviral activity similar to ACV, but at a concentration five times lower. On the other hand, PEN-containing complexes show a capability to inhibit viral replication similar to that of the sole PEN. All examined complexes induced significant microscopic alteration of cell morphology at concentrations comparable to those of PEN, except for [Pt(PEN)_2_(Me_2_phen)](NO_3_)_2_ which was less toxic compared to the other tested analogues. Differently, the ACV/PEN and Me_2_phen-containing complexes demonstrated higher antiviral activity against CMV compared to the antiviral drugs ACV or PEN [59]. On the contrary, all four tested platinum compounds were not effective against HIV. Finally, it is interesting to note that the Me_2_phen-containing complexes were generally more active with respect to the phen-containing analogues. These results could be ascribed to a different stability in the biological medium of phen and neocuproine derivatives [99].

The nucleoside tubercidin (7-deaza-adenosine), extracted from the bacterium *Streptomyces tubercidicus*, is a potent antibiotic, antiviral, and antitumor agent, but it is highly toxic against humans. It is phosphorylated by cellular kinases to the corresponding triphosphate, and incorporated into DNA/RNA, causing damage to nucleic acids functions. In addition, tubercidin takes part in several cellular processes, such as pre-mRNA processing, mitochondrial respiration, and the synthesis of purines. In the last few years, many tubercidin analogues were synthetized, with the aim to reduce its strong toxicity. The main modifications affect the *C6*, *C7*, and *C8* positions of the purine tubercidin. D’Errico et al. tested the anticancer potential of some neutral platinum(II) complexes carrying the cisplatin-like moiety linked to tubercidin through a *N*-alkyl-amide diamino spacer at the purine *C6* position. Thanks to their capability to react more quickly than cisplatin with model duplex DNA chains, it would be interesting to also evaluate the antiviral activity of such complexes [101,102,103,104,105].

Farrell et al. synthesized other platinum-nucleosides analogues having the general structural formula [PtXA_m_B_3-m_] (X= anionic ligand, A= acyclic nitrogen containing monodentate ligand, B= nucleobase derivative). They proposed and patented a method based on the administration of Pt-complexes for viral infection treatment and prevention in a host or a patient [106]. Among the examined 9-ethylguanine analogues, *trans*-[PtCl(*N7*-9-ethylguanine)(NH_3_)_2_]NO_3_ and *trans*-[PtCl(*N7*-9-ethylguanine)(NH_3_)(quinoline)]NO_3_ demonstrated protective capabilities in the host cells against HIV infection, after administration [106,107]. The affinity towards *S*-donor ligands of *trans*-[PtCl(*N7*-9-ethylguanine)(NH_3_)(quinoline)]NO_3_ complex was exploited to eject zinc from the zinc finger site of the *C*-terminal finger of the HIV-nucleocapsid protein (NCp7), which is critical for the viral life cycle [107,108]. Therefore, the ability of this platinum-nucleosides analogue to remove zinc makes it a good candidate for antiviral therapy. On the other hand, *trans*-[PtCl(*N7*-9-ethylguanine)(NH_3_)_2_]NO_3_ showed good viral inhibition at subtoxic concentration against other type of viruses, such as HCMV, VCV, HSV-1 and 2, EBV, and VZV [107].

Among nucleotide analogues directed against the RNA polymerases of RNA-dependent viruses (i.e., HCV and SARS-CoV-2), remdesivir is the only one that is FDA approved [109]. It is a monophosphoramidate prodrug of an adenosine analogue with a broad antiviral spectrum including negative-sense (filoviruses, paramyxoviruses, pneumoviruses) and positive-sense (coronaviruses) single-stranded viruses [108,110,111]. It also demonstrated to be highly effective against coronaviruses (CoVs), including MERS-Cov and SARS-CoV-2 [94,112,113,114,115,116,117,118,119,120]. Abacavir, zidovudine, lamivudine, tenofovir, stavudine, didanosine, zalcitabine, and emtricitabine are nucleoside/nucleotide analogues approved by the FDA for the treatment of HIV infections. Each of these analogues (except for tenofovir) is administered as a nucleoside and metabolized to a nucleoside triphosphate, becoming active in vivo. They lack a 3′-OH and thereby induce chain termination after being incorporated by HIV reverse transcriptase [109]. Recently, Shahabadi et al. synthetized a new fluorescent Pt-complex containing the anti-HIV didanosine drug ddI (i.e., K[PtCl(OCH_3_)_2_(ddI)] complex), with the purpose of designing a new nucleoside analogue with improved clinical efficacy. The application of spectroscopic techniques such as UV absorption, fluorescence spectroscopy, and dynamic viscosity measurements allowed the study of the interaction of K[PtCl(OCH_3_)_2_(ddI)] complex with calf thymus DNA (ct-DNA). Experiments showed that this complex can give formation of adducts with DNA through hydrogen and van der Waals interactions [121].

The discovery of “Platinblau” was attributed to Hofmann and Bugge in 1908 [122], who, in aqueous solution, reacted Ag_2_SO_4_ with [Pt(CH_3_CN)_2_Cl_2_] and isolated deep blue complexes. Interest in these platinum complexes derived from their antitumor activity, radio-sensitizing action, high water solubility, and low levels of renal toxicity [123,124]. In 1975, Rosenberg and collaborators reported on a new class of “platinum blues” (also called “Platinblau”), obtained by reacting the diaquo species of the antitumor agent cisplatin with pyrimidine nucleobases (such as uracil or thymine, as well as their *N1*-blocked derivatives, e.g., 1-methyluracil). Their DNA-binding properties and unusually high solubility make these platinum compounds particularly promising for cancer therapy, as well as bacterial and viral infections treatment. Among the studied “Platinblau” compounds, the most interesting was the *cis*-diaquodiammineplatinum(II)-uracil complex, which was demonstrated to be highly effective against the fowl pox virus (FPV), given its entrance into the cell and inhibition of the viral replication process [124].

The potential of platinum complexes in viral diseases remains an unexplored field. Nevertheless, since the discovery of the antitumor activity of platinum compounds, these molecules were studied worldwide in order to improve their efficacy and selectivity. Considering that most of the currently approved antiviral drugs are based on nucleos(t)ide analogues, the study of the ligand properties of modified nucleos(t)ides towards metal centers is an interesting promising field in the design of novel antivirals [102,121]. DNA is a primary target for many antiviral drugs because inhibition of DNA and/or RNA synthesis during viral replication is an important step, sometimes necessary, for the design of new antiviral drugs. For these reasons, the ability of some nucleos(t)ides-based platinum(II) compounds to interfere with the production, function, and/or metabolism of nucleic acids, even at low dosages, could enhance the efficacy and tolerance profiles of antiviral drugs based on modified nucleos(t)ides [59,107,124].

## 3. Cellular Processing of Platinated Nucleotides

It is well known that cisplatin, *cis*-[Pt(NH_3_)_2_Cl_2_], i.e., the most studied platinum-based antitumor drug, affects the replication of tumor cells by bonding purines into DNA in the *N7* position [72]. Such interactions seem pivotal for antitumor activity, explaining why most of the studies on cisplatin’s mechanism of action are centered on a better understanding of cisplatin–DNA interactions [125]. On the other hand, the cisplatin analogues are known to bind many biological targets (beside DNA) and for this, a comprehensive understanding of the interactions resulting in therapeutic or side effects is still in progress. The similarity with natural nucleosides makes platinum-nucleos(t)ide complexes suitable biomimetic substrates belonging to the nucleos(t)ide analogous family. Indeed, for some platinum-nucleoside compounds, cellular processing like that of the NAs was hypothesized (see Figure 2). They can act as prodrugs and, once introduced inside cells by the plasma membrane nucleoside transporters, require intracellular phosphorylation to generate their pharmacologically active triphosphate form [38]. Many investigations have focused on the study of the synthesis, structural features, and antitumor and/or antiviral biological effects of platinated nucleotides [74,79,89,126,127,128,129,130,131,132,133]. Recently, our research efforts were devoted to a deeper comprehension of the cellular metabolic mechanisms involving some model platinated nucleos(t)ides, to confirm their potential as antimetabolites. The obtained results are summarized hereafter.

### 3.1. Incorporation of Model Platinum-Nucleotide Complexes into Newly Synthesized DNA by DNA Polymerases

**Figure 11:**
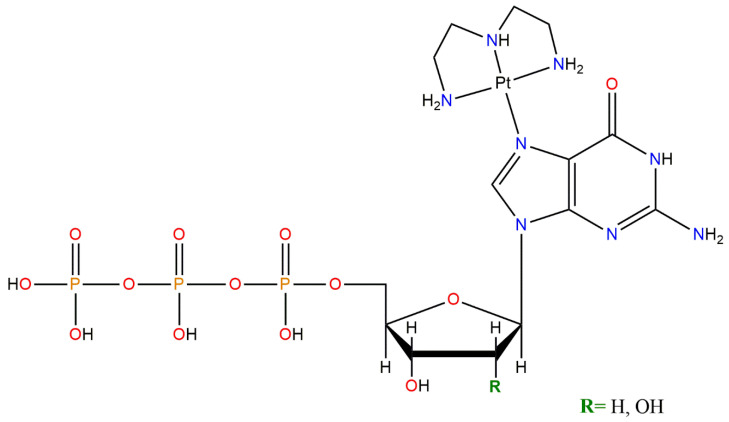
Schematic representation of the [Pt(dien)(*N*7-5′-GTP)] and [Pt(dien)(*N*7-5′-dGTP)], with R = OH and H, respectively.

Nucleotides are physiologically present in the cell cytoplasm for important processes, in particular, for the synthesis of nucleic acids [134]. A requirement to consider platinated nucleotides as antimetabolites is their recognition as substrates from the cellular enzymes, as occurs for the natural counterpart. For this purpose, we synthesized a purine–platinum complex, in the form of [Pt(dien)(5′-dGTP)], to be used as a model for cellular processing studies. The considered compound has a platinum atom attached to the diethylene-triamine tridentate ligand and to a *N7*-bonded 5′-dGTP (see Figure 11). The lack of labile chlorido ligands in its structure makes the complex unable to give mono- or bis-adducts by direct reaction with DNA, as generally occurs for cisplatin derivatives. Thus, the presence of platinum in the newly synthesized DNA can only be ascribed to the recognition by cell enzymes of the platinated nucleotide, with consequent insertion in the newly synthesized DNA, as occurs for natural nucleotides. Therefore, the [Pt(dien)(5′-dGTP)] complex can work as an antimetabolite.

**Figure 12:**
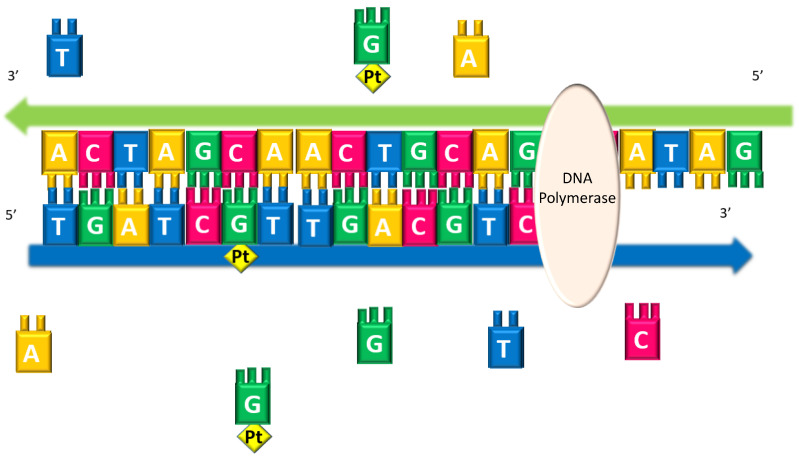
Schematic representation of the insertion mechanism of platinated nucleotides operated by DNA polymerases, during the synthesis of the complementary DNA chain, in the presence of platinated guanines in the nucleotides cellular pool.

This was recently studied by some of us, reporting clear experimental evidence that the DNA polymerases can recognize and incorporate the *N7*-platinated guanines in the in vitro and in vivo synthesis of DNA, to give platinated DNA (Figure 12) [135].

Moreover, by incorporating the metal-modified nucleotides into DNA, a possible site-specific metalation was also proposed. This possibility to obtain a polynucleotide sequence containing a nucleotide covalently linked to metal in a specific position could be of particular interest for different applications ranging from pharmacological to technological [135,136].

### 3.2. Effects of N7-Platinated Ribonucleotides on RNA Polymerases Activity

DNA and RNA strongly differ in their biological functions in relation to information storage and cellular processing [137]. A limited amount of research has focused on platinum drugs binding to RNA, rather than on the study of the stalling of transcription activities of RNA polymerases induced by platinum complexes when bonded to DNA.

We tested the *T7* RNA polymerase for the incorporation of the model compound, i.e., [Pt(dien)(*N7*-5′-GTP)] (dien = diethylenetriamine; GTP = 5′-guanosine triphosphate), into a natural RNA sequence (Escherichia coli lacZ gene encoding for β-galactosidase) by in vitro transcription [138]. The considered complex is unable to form platinum chelates with RNA. The metalated nucleotide was tested as substrate for *T7* RNA polymerase under standard in vitro transcription conditions. Our findings suggest that RNA polymerases, unlike DNA polymerases [135], are unable to incorporate the *N7*-platinated nucleotides into newly synthesized nucleic acids. This result suggests higher activation energy and selectivity for the NTPs with respect to dNTPs insertion since the nucleotide triphosphate insertion catalyzed by RNA polymerases is generally slower with respect to the analogous reaction promoted by DNA polymerases. Indeed, the RNA polymerases have lower processivity with respect to DNA polymerases and frequently abort the synthesis after the incorporation of the first few nucleotides [139,140]. The use of platinated deoxy ribonucleotides could than offer a strategy to specifically target DNA, without affecting the RNA. This class of compounds could be specifically designed and used as an alternative to the classical platinum-based drugs, with a possible reduction of both systemic toxicity and side effects, generally produced by platinum drugs for the possible direct binding to RNA.

### 3.3. Plasma Membrane Transport of Platinated Nucleos(t)ides

The uptake across the plasma membrane is one of the fundamental steps at the base of the mechanism of drugs’ pharmacological activity and resistance processes. Nucleotide analogues-induced cell toxicity is frequently limited by inefficient cellular uptake. This can determine a too-low concentration level in the cytoplasm to have a useful pharmacological activity [141]. Indeed, the ability of platinated nucleos(t)ide derivatives to mimic the natural cellular nucleos(t)ides depends on their bioavailability in cells, which is also conditioned by their efficiency of transport at the membrane cell level. Two families of transporters are involved in the natural nucleotides uptake: equilibrative transporters (ENT), which act according to the substrate concentration, and concentrative nucleotide transporters (CNT), which act against the concentration gradients in a Na^+^-dependent manner. In this frame, we experimentally studied the cellular uptake of three *N7*-platinated purines, differing in the number of phosphates carried by the nucleoside moiety and overall molecular charge. Such complexes were of the following types: [Pt(dien)(*N7*-dGuo)]^2+^ (dGuo = 2′-deoxy-guanosine), [Pt(dien)(*N7*-5′-dGMP)] (5′-dGMP = 5′-(2′-deoxy)-guanosine monophosphate), and [Pt(dien)(*N7*-5′-dGTP)]^2−^. Interestingly, from these experiments, a higher intracellular uptake for the two electrically charged [Pt(dien)(*N*7-dGuo)]^2+^ and [Pt(dien)(*N*7-5′-dGTP)]^2−^ complexes resulted, while the uptake of the neutral [Pt(dien)(*N7*-5′-dGMP)] was negligible [129]. Consistently, the electrically charged complexes [Pt(dien)(*N7*-dGuo)]^2+^ and [Pt(dien)(*N7*-5′-dGTP)]^2−^ demonstrated higher cytotoxic activity on human cervical adenocarcinoma epithelial cells (HeLa), with respect to the neutral analogue. In detail, the cell membrane transporters involved in the uptake of considered platinated nucleos(t)ides resulted in being Na^+^-dependent, which occurred for the concentrative transporters of natural nucleosides. These results suggest possible applications of the electrically charged platinated nucleos(t)ides as antiviral/antitumor drugs for more effective therapeutic strategies [142].

### 3.4. Transport and Incorporation in Mitochondrial DNA (mtDNA) of Platinated Nucleotide Analogues

Mitochondria play an important role in cell death induction [143]. Recently, we also evaluated the possibility of metalated deoxynucleotide analogues being transported into mitochondria and then incorporated into mitochondrial DNA (mtDNA) [144,145]. We first demonstrated the direct involvement of a mitochondrial carrier (*Drosophila melanogaster* thiamine pyrophosphate carrier protein, DmTpc1) in metalated purines transport [145]. DmTpc1 is a protein of the inner mitochondrial membrane that promotes mitochondrial uptake of thiamine pyrophosphate and nucleotides [146]. It was shown that the DmTpc1 transporter is able to promote the specific uptake of *N7*-platinated purines in the form of complexes [Pt(dien)(*N7*-5′-dGTP)]^2−^ (dien = diethylenetriamine; 5′-dGTP = 5′-(2′-deoxy)-guanosine triphosphate) and *cis*-[Pt(NH_3_)_2_(py)(*N7*-5′-dGTP)]^2−^ (py = pyridine), which are related to the mechanism of action of cisplatin. DmTpc1 showed a lower affinity for the two platinated purines compared to the dATP, which is the preferred substrate. However, the good affinity of DmTpc1 for platinated purines could explain the correlation between the eventual cell toxicity of platinum-bonded nucleos(t)ides and their mitochondrial import. Moreover, it was shown that a lower steric hindrance of platinated nucleos(t)ides could enhance the transport at the mitochondrial membrane level, as demonstrated by the higher uptake of complex [Pt(dien)(*N7*-5′-dGTP)]^2−^ compared to *cis*-[Pt(NH_3_)_2_(py)(*N7*-5′-dGTP)]^2−^ [145]. Similar results were obtained by Lunetti et al. [144], even with the affinity of the human mitochondrial deoxynucleotide carrier (DNC) and its possible involvement in the mitochondrial uptake of platinated nucleos(t)ides. Kinetic characterization, followed by ICP-AES (inductively coupled plasma atomic emission spectroscopy), showed that the [Pt(dien)(*N7*-5′-dGTP)]^2−^ was highly and selectively accumulated into proteoliposomes reconstituted with the purified recombinant DNC, demonstrating the possible involvement of this transporter in the transport of platinated nucleotides across the inner mitochondrial membrane. Even in this case, the *cis*-[Pt(NH_3_)_2_(py)(*N7*-5′-dGTP)]^2−^ complex is transported less efficiently, probably due to its higher steric hindrance. Finally, experiments in organello were permitted to observe the incorporation of the [Pt(dien)(*N7*-5′-dGTP)] complex into the *mt*DNA of freshly prepared rat liver mitochondria by DNA polymerase γ, in a time-dependent manner [144]. Thus, the DNC and the mitochondrial polymerase γ could be directly involved in the cytotoxicity mechanism of platinated nucleobases, suggesting the possibility of applying metalated nucleoside analogues-based drugs in the treatment of a wide range of diseases.

## 4. Conclusions

Metalated nucleos(t)ides are a new class of potential antitumor/antiviral drugs that merge the antitumor and/or antiviral properties of metal compounds and of nucleos(t)ides analogues antimetabolites in the same compound. The study of this new class of promising antitumor/antiviral compounds is still an unexplored field. This review aimed to collect all the scientific research concerned with the evidenced properties as antitumor/antiviral agents of platinated nucleotide complexes.

**Figure 13:**
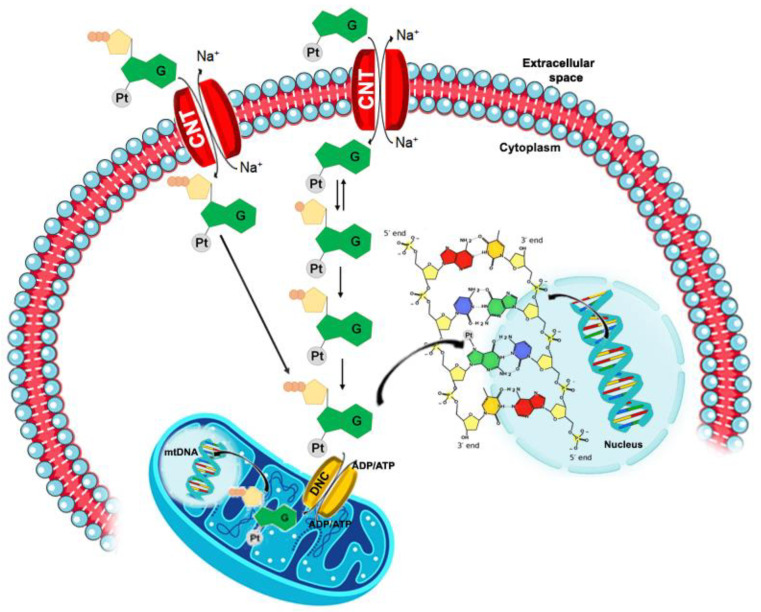
Proposed mechanism of action of platinated nucleos(t)ides.

As previously discussed, platinum-bonded nucleos(t)ides, considered as nucleoside analogues, could act as biomimetic substrates and be selectively internalized and/or processed in cells. Platinated nucleos(t)ides could follow a pathway similar to that of other nucleoside analogues-based drugs. This entails that after their eventual uptake by membrane transporters (cellular and/or mitochondrial) and after phosphorylation, they could also be recognized by nuclear and/or mitochondrial DNA polymerases and incorporated into newly synthesized DNA (see Figure 13). This could induce cancer cells or viruses’ death.

In conclusion, anticancer and/or antiviral metalated nucleotide analogues could act as a new generation of metal-based drugs, characterized by more specific cellular targets and fewer side effects. The possibility of controlled mechanisms of both cell uptake and cellular processing could be a relevant improvement with respect to common metal-based drugs. Indeed, these latter drugs generally undergo a wide set of mechanisms of action and toxicity often not easily defined and controlled. This situation is at the basis of low specificity and relevant side effects generally shown by metal-based drugs. Low-specificity problems are particularly felt for platinum antitumor drugs, where the reduction and control of side effects remains one of the main issues to be addressed. The studies reviewed here suggest considering platinum-nucleotide adducts as a potential new class of pharmacologically active antitumor/antiviral drugs and metal-based antimetabolites, to be further explored.

## Figures and Tables

**Table 1:** Selection of clinically used antitumor and antiviral nucleos(t)ide analogues (NAs) antimetabolites.

Antitumor Antimetabolites	Molecular Formula	Clinical Use	References
Purine analogues			
6-Mercaptopurine	C_5_H_4_N_4_S	Acute lymphocytic and acute myelogenous leukemias and small cell non-Hodgkin’s lymphoma	[9,10]
Fludarabine	C_10_H_12_FN_5_O_4_	Chronic lymphocytic leukemia and low-grade lymphomas	[11]
Cladribine	C_10_H_12_ClN_5_O_3_	Hairy cell leukemia and low-grade lymphomas	[12]
Clofarabine	C_10_H_11_ClFN_5_O_3_	Pediatric acute leukemia	[13]
Pyrimidine analogues			
5-Fluorouracil	C_4_H_3_FN_2_O_2_	Head and neck, colon, breast, esophageal, stomach, pancreas, premalignant skin	[14]
Cytarabine	C_9_H_13_N_3_O_5_	Acute lymphocytic leukemia, non-Hodgkin’s lymphoma, and acute myelogenous leukemia	[15]
5-Azacytidine	C_8_H_12_N_4_O_5_	Myelodysplasia	[16]
Folate analogues			
Methotrexate	C_20_H_22_N_8_O_5_	Acute lymphocytic leukemia, osteosarcoma, bladder cancer, head, neck, and lung cancer, breast	[17]
Pemetrexed	C_20_H_21_N_5_O_6_	Mesothelioma and lung cancer	[18]
**Antiviral antimetabolites**			
Purine analogues			
Aciclovir	C_8_H_11_N_5_O_3_	Chickenpox, shingles, herpes virus	[19]
Famciclovir	C_14_H_19_N_5_O_4_	Shingles	[20]
Ganciclovir	C_9_H_13_N_5_O_4_	Cytomegalovirus (CMV) infection of the eyes in people whose immune system is compromised	[21]
Penciclovir	C_10_H_15_N_5_O_3_	Herpes simplex virus infections around the mouth (cold sores)	[22]
Valaciclovir	C_13_H_20_N_6_O_4_	Herpes virus infections, shingles, and herpes simplex in adults	[23]
Valganciclovir	C_14_H_22_N_6_O_5_	Cytomegalovirus (CMV) retinitis in people who have acquired immunodeficiency syndrome (AIDS)	[24]
Vidarabine	C_10_H_15_N_5_O_5_	Acute keratoconjunctivitis and recurrent epithelial keratitis due to herpes simplex virus types l and 2.	[25]
Adefovir	C_8_H_12_N_5_O_4_P	Chronic (long-term) hepatitis B infection	[26]
Didanosine	C_10_H_12_N_4_O_3_	Human immunodeficiency virus (HIV) infection	[27]
Entecavir	C_12_H_15_N_5_O_3_	Hepatitis B virus	[28]
Tenofovir	C_9_H_14_N_5_O_4_P	HIV infection in adults and children	[29]
Pyrimidine analogues			
Cidofovir	C_8_H_14_N_3_O_6_P	Cytomegaloviral retinitis in people with acquired immunodeficiency syndrome (AIDS)	[30]
Trifluridine	C_10_H_11_F_3_N_2_O_5_	Keratoconjunctivitis and recurrent epithelial keratitis due to herpes simplex virus, types 1 and 2	[31]
Emtricitabine	C_8_H_10_FN_3_O_3_S	Human immunodeficiency virus type 1 (HIV-1) (in combination with at least one other HIV drug)	[32]
Lamivudine	C_8_H_11_N_3_O_3_S	HIV-1 infection, hepatitis B (HBV) virus infection	[33]

## Data Availability

No new data were created.
